# Impact of Genetic Variants on the Individual Potential for Body Fat Loss

**DOI:** 10.3390/nu10030266

**Published:** 2018-02-26

**Authors:** Soyeon Cha, Joon Ho Kang, Jae-Hak Lee, Jinki Kim, Heewon Kim, Yoon Jung Yang, Woong-Yang Park, Jinho Kim

**Affiliations:** 1 Samsung Genome Institute, Samsung Medical Center, Gangnam-gu, Seoul 06351, Korea; scha@ncsu.edu (S.C.); kangx342@gmail.com (J.H.K.); noonolee@gmail.com (J.-H.L.); wonniey@gmail.com (H.K.); 2 Department of Molecular Cell Biology, Sungkyunkwan University School of Medicine, Suwon 16419, Gyeonggi-do, Korea; 3 Software R&D Center, Samsung Electronics, Hwaseong 18448, Gyeonggi-do, Korea; jk.neo.kim@samsung.com; 4 Department of Food and Nutrition, Dongduk Women’s University, Seoul 02748, Korea; yjyang@dongduk.ac.kr

**Keywords:** GWAS, BMI, body fat, obesity, diet, health, exercise, genetic risk score

## Abstract

The past decade has witnessed the discovery of obesity-related genetic variants and their functions through genome-wide association studies. Combinations of risk alleles can influence obesity phenotypes with different degrees of effectiveness across various individuals by interacting with environmental factors. We examined the interaction between genetic variation and changes in dietary habits or exercise that influences body fat loss from a large Korean cohort (*n* = 8840). Out of 673 obesity-related SNPs, a total of 100 SNPs (37 for carbohydrate intake; 19 for fat intake; 44 for total calories intake; 25 for exercise onset) identified to have gene-environment interaction effect in generalized linear model were used to calculate genetic risk scores (GRS). Based on the GRS distribution, we divided the population into four levels, namely, “very insensitive”, “insensitive”, “sensitive”, and “very sensitive” for each of the four categories, “carbohydrate intake”, “fat intake”, “total calories intake”, and “exercise”. Overall, the mean body fat loss became larger when the sensitivity level was increased. In conclusion, genetic variants influence the effectiveness of dietary regimes for body fat loss. Based on our findings, we suggest a platform for personalized body fat management by providing the most suitable and effective nutrition or activity plan specific to an individual.

## 1. Introduction

The advent of genome-wide association studies (GWAS) has enabled the discovery of thousands of genetic variants that contribute to human diseases in the last decade [1]. Multiple risk alleles identified from GWAS have been useful in predicting the risk of patients developing various diseases, including metabolic syndrome, hypertension, hyperlipidemia, type 2 diabetes mellitus, heart disease, cardiovascular disease, and other comorbidities [2,3,4]. Obesity-related genetic variants are major contributors to these complex diseases and have thus been extensively studied. In 2007, the first single nucleotide polymorphisms (SNPs) mapped to the fat mass and obesity-associated (*FTO*) gene region were found to be associated with body mass index (BMI) [5,6,7,8,9,10]. Subsequent identification of other SNPs has accelerated the characterization of the effects of different variant combinations on obesity phenotype, as well as their interactions with environmental factors, such as dietary habits, lifestyle, and physical activity [11,12,13,14,15,16]. In addition, the market for the direct-to-consumer (DTC) genomic testing has emerged [17,18]. Personal genomics companies, such as Pathway Genomics, DNA Fit, 23andMe, Navigenics, and Helix Health, have offered personalized genetic reports that provide information on future medical risks, fitness, and athletic ability, as well as recommendations on diet, skin care, or lifestyle. However, the U.S. federal drug administration (FDA) has halted the continuation of these services because of the lack of adequate scientific evidence, although such services have been relaunched recently. Thus, detailed investigations that utilize genetic screening tools are required to establish more reliable personalized health care programs.

In this study, we aimed to identify the genetic variants that influence an individual’s potential for body fat loss. Our study was motivated by the results of previous studies, which demonstrated that each individual has a genetic different predisposition and can thus benefit from a specific diet and exercise regime [19,20,21,22,23,24]. An individual’s response to changes in nutrient intake or physical activity may depend on the number of risk alleles. Thus, we tested the associations between the body fat loss and SNPs that interact with either reductions in energy intake or increase in exercise. A set of selected SNPs were used to score each individual’s sensitivity to body fat loss through the modulation of environmental variables, including carbohydrate intake, fat intake, total calorie intake, and exercise status. Our study explores the following novel aspects: (1) assessment of the applicability of obesity-related SNPs to Koreans; (2) evaluation of the interaction between genetic and environmental factors that influence the extent of body fat loss; and (3) incorporation of changes in total calorie intake, carbohydrate intake, and fat intake into the model using data from a large Korean cohort obtained by administering food frequency questionnaires (FFQ).

## 2. Methods

### 2.1. Data Source

A large Korean cohort was obtained from the Korea Association REsource (KARE) [25], the first project of Korean Genome and Epidemiology Study (KoGES). A total of 8840 participants were surveyed biannually from 2001 (baseline) to 2013 (seventh follow-up) in two cities (Ansung and Ansan). Collected dataset comprised a total of 503 items for 16 categories, including general characteristics, drinking history, smoking history, physical activities, drug history, past medical history, treatment history, family history of disease, reproductive factors, body composition, vital signs, chest X-ray, clinical examination, anthropometry, dietary habit, and feeding frequency. Among these, items that were used as independent variables in our study included food frequency questionnaire (FFQ), which was available only for the first and third follow-ups (*n* = 4293), and intensive exercise status, which was available from the third to seventh follow-up (*n* = 3343). In addition, a subset of SNPs was extracted from whole-genome sequencing data of the cohort using PLINK v1.07 software [26,27]. The pre-defined subset comprising 673 SNPs that are related to obesity and waist-to-hip ratio was obtained from the GWAS catalog database.

### 2.2. Ethics Statement

The protocol of the clinical study was approved by the Institutional Review Board (2016-07-029) of the Korea Centers for Disease Control and Prevention and all experiments were conducted in accordance with the approved guidelines. The data was obtained from the Korean Genome and Epidemiology Study (KoGES; 4851-302). Written informed consent was obtained from all participants.

### 2.3. Linear Regression Model and Genetic Risk Score

**Figure 1:**
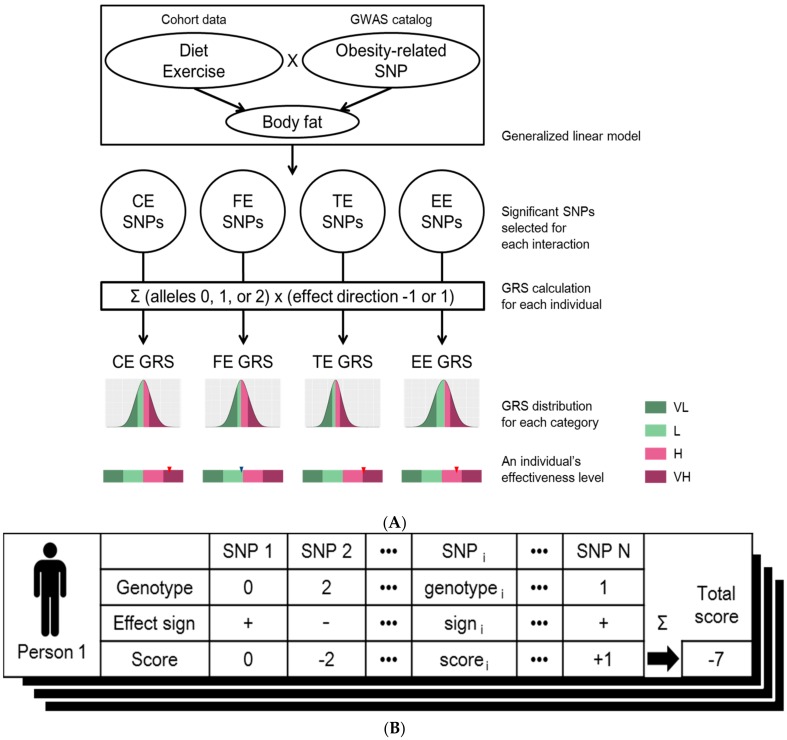
(**A**) Workflow for designing genome-based personalized health care program. Among the set of SNPs related to obesity under GWAS catalog, SNPs of which has significant interaction effects for diet or exercise were assigned to four different effectiveness categories using generalized linear model: effectiveness of “carbohydrate intake changes” (CE), effectiveness of “fat intake changes” (FE), effectiveness of “total calories intake changes” (TE) or “effectiveness of exercise” (EE). Genetic risk scores (GRS) of four effectiveness categories were calculated per individual. The distributions of GRSs for each of four effectiveness classes were generated using the KoGES cohort. The degree of effectiveness was defined as “very low” (VL; smaller than the 25th percentile GRS), “low” (L; larger than or equal to the 25th percentile and smaller than the 50th percentile GRS), “high” (H; larger than or equal to the 50th percentile and smaller than the 75th percentile GRS), and “very high” (VH; larger than or equal to the 75th percentile GRS) for all of four GRS distributions; (**B**) Calculation of genetic risk score (GRS). The GRS of an individual is calculated based on the genotypes of the selected SNPs (column names of the table). The score of each SNP (3rd row in the table) was calculated by multiplying the genotype that is the number of risk alleles of each SNPs (1st row in the table) by the effect sign (2nd row in the table) which is determined by the sign of estimated coefficient of the corresponding interaction term from the linear model. These scores are summed over all the selected SNPs to generate the final effectiveness score of the individual (denoted as “Total score” at the right corner of the table). This process was applied to all of the individuals, as well as all of the four categories: “effectiveness of carbohydrate intake changes” (CE), “effectiveness of fat intake changes” (FE), “effectiveness of total calories intake changes” (TE), and “effectiveness of exercise” (EE). Thus, a total of four GRS distributions were generated.

The overall workflow of our approach is illustrated in Figure 1A. We started from identifying SNPs that interact with the nutrient intake and exercise status on body fat change. To evaluate changes in body fat mass that were influenced by the interactions between genetic risk factors and changes in dietary habits or exercise status at individual SNP level, we adopted a generalized linear model to test each SNP. The additive genetic effect was modeled as 0, 1, or 2, which encode the number of copies of the minor alleles. After adjusting for covariates, including gender and age, we examined (1) the effects of the interaction between a SNP and change in either carbohydrate intake (ΔC; gram) or fat intake (ΔF; gram) on body fat mass (kilogram); (2) effect of interaction between a SNP and changes in daily calorie intake (gram) on changes in body fat mass (kilogram); and (3) the main effect of SNP on changes in body fat mass (kilogram) corresponding to changes in exercise status. Model (1) was adjusted for changes in total calorie intake (ΔTC) to measure the sole effect of nutrients. Furthermore, the exercise status of the cohort obtained from the third to seventh follow-up studies was measured as binary variables, with 1 for the response “no” and the value 2 for “yes” to the question “Please check if you have regularly exercised enough for sweat.” To regularize this variable with uneven missing values across individuals and time points, the difference in body fat mass was measured between two consecutive time points from the time point with response 2 to that with response 1, regardless of time directionality. Changes in body fat mass over time were averaged for each individual and used as the dependent variable Δbody fat * in model (3). The following three models were previously described:

(1) $$\Delta \mathrm{body}\;\mathrm{fat}\;=\;\mathrm{gender}\;+\;\mathrm{age}\;+\;\Delta \mathrm{TC}\;+{\;\mathrm{SNP}}_{i}\;+\;\Delta C\;+\;\Delta F\;+{\;\beta }_{c\;}\Delta C\;\times {\;\mathrm{SNP}}_{i}\;+{\;\beta }_{f\;}\Delta F\;\times {\mathrm{SNP}}_{i}$$

(2) $$\Delta \mathrm{body}\;\mathrm{fat}\;=\;\mathrm{gender}\;+\;\mathrm{age}\;+\;\Delta \mathrm{TC}\;+{\;\mathrm{SNP}}_{i}\;+{\;\beta }_{\mathrm{TC}}\Delta \mathrm{TC}\;\times {\;\mathrm{SNP}}_{i}\;$$

(3) $$\Delta \mathrm{body}\;\mathrm{fat}*\;=\;\mathrm{gender}\;+\;\mathrm{age}\;+{\;\beta }_{E}{\;\mathrm{SNP}}_{i}$$

Statistical analyses were performed using the standard package glm in R software (http://www.r-project.org/) for model fitting.

Basically, SNPs with interaction effects “ΔC × SNP” or “ΔF × SNP” *p*-values lower than 0.05 were selected to calculate genetic risk scores for “effectiveness of carbohydrate intake changes” (CE) or “effectiveness of fat intake changes” (FE), respectively. Similarly, SNPs with interaction effect “ΔTC × SNP” in model (2) or with main effect in model (3) and met the threshold of 0.05, were used for defining “effectiveness of total calories intake changes” (TE) or “effectiveness of exercise” (EE), respectively. The SNAP tool was used to identify SNPs in linkage disequilibrium (LD) for each category (http://www.broad.mit.edu/mpg/snap/) [28]. If the scores of pairwise LD among a set of SNPs were higher than 0.7, only one SNP with the term coefficient having smallest *p*-value was retained. This process was performed for each the four categories, CE, FE, TE, and EE.

The final set of SNPs was used to count the number of risk alleles of an individual. Each of the SNPs was assigned the coefficient sign of the corresponding term (β_f_, β_F_, β_TC_) in the linear model. On the other hand, the sign was reversed for the term β_E_ because a positive coefficient represents a positive genetic effect of exercise onset on weight gain; however, we aimed to increase the TE score as a result of effective weight loss through exercise. Next, each individual’s genetic risk score was calculated by multiplying the number of risk alleles for each SNP by the assigned sign and summing over all SNPs (Figure 1B). The steps were performed for each of the four categories to generate four genetic risk scores (GRS) per individual. For all four GRS distributions, the degrees of effectiveness were defined as “very low” (VL; smaller than the 25th percentile GRS), “low” (L; larger than or equal to the 25th percentile and smaller than the 50th percentile GRS), “high” (H; larger than or equal to the 50th percentile and smaller than the 75th percentile GRS), and “very high” (VH; larger than or equal to the 75th percentile GRS). Thus, each individual can be assigned to one of four classes (VL, L, H, and VH) for each of the four categories (CE, FE, TE, and EE).

## 3. Results

### 3.1. Characteristics of the Cohort

The KoGES study included a total of 8840 participants. Of these 4293 (2070 males and 2223 females) participants had complete FFQ data (baseline and third follow-up) and body composition measurements and were subsequently analyzed for changes in dietary habits (Table 1). Individuals who experienced drastic changes in exercise status at least once over five follow-ups (third to seventh) were included in the study on changes in exercise regime (*n* = 3343; 1516 males and 1827 females). The mean ages were about 51 and 55 years for the dietary and exercise study, respectively. The mean difference in daily carbohydrate intake between the baseline and the third follow-up was −18.40 g, while the mean differences in fat and total calorie intake were −4.71 g and −147.12 g, respectively. The mean differences in BMI and body fat mass were −0.14 kg/m^2^ and −0.42 kg, respectively. To investigate the effects of exercise, the differences in mean body fat mass between consecutive time points for those who responded with either yes or no to significant increase in exercise status are summarized in Table 1. To incorporate temporal data points from the third to seventh follow-ups, the change in body fat mass for each individual was measured from the point of response “yes” to that of response “no” regardless of time directionality. The differences were averaged, and the mean of averaged body fat mass difference was −0.08 kg.

### 3.2. SNP Selection by Generalized Linear Model

Generalized linear model (GLM) was used for single SNP analysis of 673 obesity-related SNPs. Results revealed a total of 111 SNPs having significant interactions with changes in diet or exercise to influence body fat mass (α = 0.05) (Supplementary file). Eighteen SNPs were in linkage disequilibrium (LD), and the SNP with the smallest *p*-value for each LD group was selected as the representative SNPs for each group, leaving a total of seven SNPs. The number of SNPs that were included in the final set of SNPs used for quantifying the effectiveness of changes in carbohydrate intake (CE), fat intake (FE), total calorie intake (TE), and exercise onset (EE) were 37, 19, 44, and 25, respectively (Table 2). CE and TE (*n* = 14) showed the highest number of SNPs that were common between any two categories, whereas only 0 to 2 SNPs were common in other sets (Figure S1). The minor allele frequencies ranged from 0.01 to 0.49 (Figure S2). Table 3 shows the top ten SNPs sorted by *p*-value and their corresponding coefficient estimates from GLM and matched gene names. For the diet study, under the categories CE, FE, and TE, the “Peroxisome proliferator-activated receptor gamma” (PPARG) gene showed the most number of mapped SNPs (*n* = 16) and was highly ranked with a low *p*-value. In addition, results suggested the interaction of “angiotensinogen” (AGT; seven SNPs mapped) with all four categories. Moreover, “Apolipoprotein A2” (APOA2; four SNPs mapped) interacted with all categories except for FE. Each of the 41 SNPs was uniquely matched to a gene, whereas 32 SNPs were mapped to intergenic regions.

### 3.3. GRS Classification and Effectiveness of Dietary Regime

**Figure 2:**
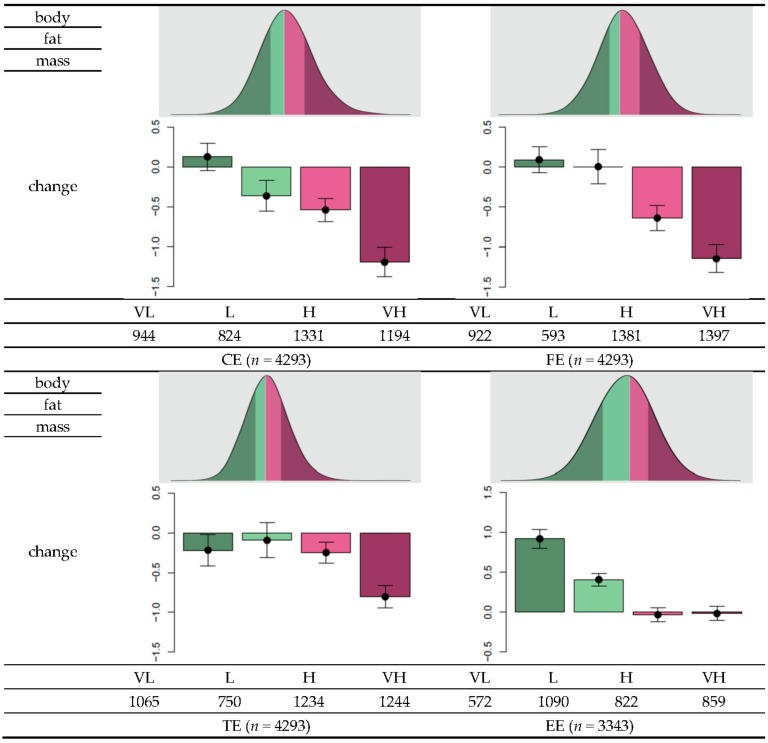
Distribution of genetic risk scores (GRS) and within-group changes in body fat as a function of changes in diet or exercise regime. Each individual’s GRS can be calculated based on the final set of SNPs to measure changes in carbohydrate intake (CE), fat intake (FE), total calorie intake (TE), and exercise status (EE). To determine the effectiveness of body fat loss, “very low” (VL) was defined to range from the minimum of GRS and the value less than the 25th percentile. The “low” (L) level ranges from the value larger than or equal to the 25th percentile and 50th percentile, “high” (H) includes values larger than or equal to the 50th percentile and less than the 75th percentile, and “very high” (VL) corresponds to values larger than or equal to the 75th percentile. Among individuals with greater than 75 g reduction in carbohydrate intake, the mean change in body fat in individuals grouped to CE-VL was 0.13, −0.37 for the group CE-L, −0.54 for the CE-H group, and −1.19 for the CE-VH group. Among individuals with at least 13 g reduction in fat intake, the mean changes in body fat were −0.22, −0.095, −0.25, and −0.80 for the groups FE-VL, FE-L, FE-H, and FE-VH, respectively. Among individuals with at least 478 kcal reduction in total calorie intake, the mean changes in the body fat were 0.093, 0.0055, −0.64, and −1.14 for the groups TE-VL, TE-L, TE-H, and TE-VH, respectively. Among individuals who experienced exercise onset, the mean changes in body fat were 0.92, 0.41, −0.033, and −0.018, respectively.

Based on the sign of the estimated coefficient, the final set of SNPs was assigned with a value of either −1 or 1 (Table 2). The genetic risk score (GRS) was calculated as the total number of risk alleles of a SNP multiplied by the assigned sign. The GRS data approximately followed a normal distribution. Descriptive statistics of the GRS distribution are summarized in Table 4. The GRS means and standard deviations were 0.43 ± 3.81, 1.4 ± 2.5, 5.4 ± 4.2, and 1.5 ± 3.1 for CE, FE, TE, and EE, respectively. To investigate changes in body fat in relation to the GRS, individuals were stratified into four groups, namely, very low (VL), low (L), high (H), and very high (VH), for each category (Figure 2). Overall, as the effectiveness level increased from VL to VH, body fat reduction tended to be higher according to either reduction in nutrient intake or exercise onset. For example, for individuals with greater than 75 g reduction in carbohydrate intake (first quantile of the distribution of changes in carbohydrate intake), the mean changes in body fat were 0.13, −0.37, −0.54, and −1.19, for the CE-VL, CE-L, CE-H, and CE-VH groups, respectively. For individuals with greater than 13 g reduction in fat intake (first quantile in the distribution of changes in fat intake) the mean changes in body fat were −0.22, −0.095, −0.25, and −0.80 for the groups FE-VL, FE-L, FE-H, and FE-VH, respectively. In individuals with greater than 478 kcal reduction in total calorie intake (first quantile in the distribution of changes in total calorie intake), the mean changes in body fat were 0.093, 0.0055, −0.64, and −1.14 for the groups TE-VL, TE-L, TE-H, and TE-VH, respectively. Among individuals who experienced exercise onset, the corresponding mean changes in body fat were 0.92, 0.41, −0.033, and −0.018. Thus, these results suggest that individuals belonging to the VH group can benefit more from reduced nutrient intake or more rigorous exercise to increase body fat loss compared to individuals classified under lower levels.

### 3.4. Personalized Genome-Based Health Care

**Figure 3:**
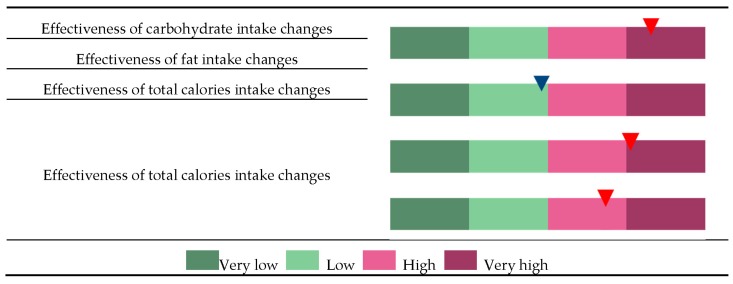
Classification of an individual to the very low (VL), low (L), high (H), and very high (VH) groups and to each of the four categories, changes in effectiveness of carbohydrate intake (CE), changes in effectiveness of fat intake (FE), changes in effectiveness of total calories intake (TE), and changes in effectiveness of exercise status (EE). Each individual’s GRS was calculated based on a set of SNPs that were selected to represent risk factors for each of the categories CE, FE, TC, and EE. In this example, the individual was classified as “CE-VH” because the GRS for CE is higher than the 75th percentile in the distribution of population GRS. Similarly, a GRS between the 25th and 50th percentile in the FE distribution, higher than the 75th percentile in the TE distribution, or between the 50th and 75th percentile in the EE distribution will be classified under FE-L, TE-VH, or EE-H, respectively.

As described above, the effectiveness of body fat loss can be determined based on the quantile of and individual’s GRS (Figure 3). For example, individuals assigned under CE-VH, FE-L, TE-VH, and EE-H groups are potentially more sensitive to changes in carbohydrate intake than fat intake but are also highly responsive to total calorie intake and exercise onset. Thus, these patients are advised to undergo intensive exercise regime and to monitor total calorie intake, particularly the proportion of carbohydrates in the diet.

## 4. Discussion

Dietary habits worldwide have become westernized, and the number of individuals diagnosed with obesity and weight control issues has continued to increase. Recent studies have uncovered the association among obesity, genetic factors, and dietary habit or physical activity. They include genetic loci contributing to dietary habit on obesity, the effects of gene-environment interactions on obesity, and modifying effects of dietary habit on the association between genetic factors and obesity [29,30,31,32,33,34,35,36,37]. As rising life expectancy, the biological insights about obesity from previous studies led to present the need for the personalized diet. Here, in this paper, we proposed a computational platform to provide the personalized guide to improve health status.

The identified SNPs may be functionally related to the metabolism of nutrients in the body (Table S1). Indeed, many of the SNPs we identified have been reported to be associated with metabolic syndrome or insulin resistance as a consequence of metabolic malfunction. In our study, 16 SNPs at *PPARG* related to CE, FE, TE, and EE were identified. PPARG is involved in lipid and glucose homeostasis [38,39], decreased plasma leptin level [40], and insulin resistance [41,42]. Especially, *PPARG* rs17793951 and rs2920502 are associated with insulin resistance [43] and the higher risk of metabolic syndrome by regulating the expression of adiponectin [44]. We also identified ARAP1 rs11603334, a genetic polymorphism associated with carbohydrate and fat intake. Kulzer et al. reported that it increases the expression of ARAP1 in human pancreatic islets, which can contribute to type 2 diabetes susceptibility [45].

Considering the potential inaccuracies of the cohort data, false positives might be present in the set of selected SNPs. Instead of conducting rigorous measurements of daily calories intake, FFQ estimates the average daily food items in list and weekly or yearly feeding frequencies. Thus, errors in the data could have arisen from subjective judgment on the day of administering the questionnaire or could have been influenced by the most recent memories of eating behavior. In addition, a fixed list of foods ignores other variable food items consumed by different individuals. Not only the FFQ estimates but also the questionnaire that used binary variables and self-reported answers for physical activity also includes limitations. The binary variable “exercise status” as the answer to the questionnaire “Please check if you do sweaty exercise regularly” could not differentiate precisely on the varied degree of physical activity, for example, low-, intermediate-, and high-intensity activities. Also, self-reported answers system has several limitations. The answer can be easily biased by many factors including individual’s mood at the time of filling out the questionnaire or resistance to answering the private question. Additionally, it is not feasible for a subject to remember the exact days of exercise in the question of “Please check if you do sweaty exercise regularly”. Furthermore, the questionnaires were administered two years apart, during which unobserved environmental factors could have influenced body fat loss.

Another limitation of the study is lack of independent validation data where the effectiveness of the proposed strategy can be evaluated. Internal validation analysis demonstrated the usefulness of the approach, but to rigorously evaluate the effect size of each group while reserving statistical power of the training dataset, an independent cohort data would be needed. In this regard, we plan to conduct prospective interventional experiments by grouping individuals into either exercise or dietary group, which are further divided into either low carbohydrate or low-fat diet groups. Also, to obtain more precise physical activity data from subjects, wearable devices will be distributed to the subjects. By applying our model to an independent data set, we expect to overcome limitations and validate the algorithm and update the SNP set that influences diet efficacy.

## 5. Conclusions

To develop an efficient strategy for effectively reducing body fat, we proposed a GWAS procedure consisting of the following steps: SNP selection by testing the interaction between genetic and environmental factors using a linear model; calculation of scores as the sum of the number of risk alleles over a set of selected SNPs; and allocation of individuals to an appropriate body fat loss regime in response to the altered environmental factor. Our proposed approach could ultimately provide an effective genome-based personalized diet strategy by determining the nutritional component that requires modulation or the degree of exercise effectiveness for a particular individual.

This research focused on the reduction of body fat mass in a whole body. One direction for further development of the study might be considering body fat distribution or metabolic health. Recent studies suggested that metabolic health is an independent factor to predict cardiovascular risk [46,47]. We expect a similar approach will be effective to develop a personalized strategy to turn metabolically unhealthy people to metabolically healthy ones, which may have a more significant impact on public health.

## Figures and Tables

**Table 1:** Characteristics of the KoGES cohort. The sample size for each gender and mean ± SD along with sample size in parenthesis for other categories are presented.

Diet		Exercise			
Male (*n*)	2070	Male (*n*)			1516
Female (*n*)	2223	Female (*n*)			1827
Age (years)	50.84 ± 8.54	Age (years)			55 ± 8.29
Carbohydrate intake at baseline (g)	343.3 ± 114.21	Body fat change according to exercise status change	(3rd to 4th)	Stopped	0.27 ± 2.16 (653)
Carbohydrate intake at 3rd follow-up (g)	324.89 ± 94.94			Started	−0.05 ± 2.24 (741)
Carbohydrate intake change (g)	−18.4 ± 132.38		(4th to 5th)	Stopped	0.07 ± 2.22 (650)
Fat intake at baseline (g)	33.25 ± 21.08			Started	−0.09 ± 2.28 (675)
Fat intake at 3rd follow-up (g)	28.54 ± 18.64		(5th to 6th)	Stopped	0.29 ± 2.15 (773)
Fat intake change (g)	−4.71 ± 22.1			Started	0.06±2.12 (616)
Total calories intake at baseline (g)	1960.19 ± 688.77		(6th to 7th)	Stopped	1.29 ± 2.37 (569)
Total calories intake at 3rd follow-up (g)	1813.06 ± 581.05			Started	1.3 ± 2.43 (662)
Total calories intake change (g)	−147.12 ± 758.94	Averaged body fat change over times	Exercise status change between any two consecutive time points irregardless of time directionality		−0.08 ± 2.11 (3343)
BMI at baseline (kg/m^2^)	24.48 ± 3				
BMI at 3rd follow-up (kg/m^2^)	24.35 ± 2.95				
BMI change (kg/m^2^)	−0.14 ± 1.2				
Body fat mass at baseline (kg)	16.86 ± 5.29				
Body fat mass at 3rd follow-up (kg)	16.44 ± 5.26				
Body fat mass change (kg)	−0.42 ± 2.57				

**Table 2:** The number of SNPs assigned with −1 or +1 for each category. The finally subset of SNPs were assigned to either negative (−1) or positive (+1) effect groups based on the sign of the estimated coefficient of the corresponding term in the linear model. As shown in the table, CE, FE, TE, and EE comprised 37 SNPs (18 negative and 19 positive), 19 SNPs (seven negative and 12 positive), 44 SNPs (14 negative and 30 positive), and 25 SNPs (12 negative and 13 positive), respectively.

	CE	FE	TC	EE
−1	18	7	14	12
1	19	12	30	13

**Table 3:** Top ten SNPs identified by the generalized linear model (GLM; sorted by *p*-value). The SNPs identified from “ΔC × SNP” in model (1), from “ΔF × SNP” in model (1), from “ΔTC × SNP” in model (2), and from “SNP” in model (3) were used to determine polygenic effectiveness for changes in carbohydrate intake (CE), fat intake (FE), total calorie intake (TE), and exercise status (EE), respectively. For polymorphisms in linkage disequilibrium (LD), only SNPs with the smallest *p*-values were selected to represent each group; this procedure was performed for each category. The coefficient estimates and *p*-values were obtained from a single SNP analysis using GLM.

Category	SNP	Estimate	*p*-Value	Gene Name
CE	rs13077495	0.006	1.90 × 10^−5^	PPARG
CE	rs206936	−0.0018	0.00016	RPS10-NUDT3
CE	rs17793951	0.0036	0.00064	PPARG
CE	rs189428681	0.0066	0.0015	PPARG
CE	rs7578465	−0.0016	0.0019	ALK
CE	rs191018871	0.0062	0.0021	PPARG
CE	rs7920888	−0.0015	0.0043	intergenic
CE	rs13041126	−0.0015	0.0052	intergenic
CE	rs6782178	0.0023	0.0056	PPARG
CE	rs6810295	0.0014	0.006	OSBPL10
FE	rs2237892	−0.01	0.00067	KCNQ1
FE	rs1413020	0.012	0.0062	intergenic
FE	rs2972165	0.029	0.0072	PPARG
FE	rs2389438	0.017	0.011	intergenic
FE	rs2938398	0.0091	0.013	PPARG
FE	rs11122577	−0.0077	0.014	AGT
FE	rs11142387	0.0074	0.019	intergenic
FE	rs7551318	−0.027	0.02	intergenic
FE	rs4243830	−0.008	0.022	PLEKHG5
FE	rs4144743	−0.0066	0.023	intergenic
TE	rs2972164	0.00056	2.50 × 10^−5^	PPARG
TE	rs11142387	0.00032	4.10 × 10^−5^	intergenic
TE	rs13077495	0.0009	5.70 × 10^−5^	PPARG
TE	rs206936	−0.00029	0.0001	RPS10-NUDT3
TE	rs67216730	0.00062	0.00044	PPARG
TE	rs2920502	0.00026	0.00059	PPARG
TE	rs7578465	−0.00025	0.0014	ALK
TE	rs13041126	−0.00022	0.0032	intergenic
TE	rs12150665	0.00021	0.0047	GGNBP2
TE	rs4327120	0.00024	0.0048	intergenic
EE	rs4432245	−0.14	0.0065	EIF2AK4
EE	rs10929925	−0.14	0.0065	intergenic
EE	rs4762	−0.21	0.013	AGT
EE	rs7164727	0.15	0.013	intergenic
EE	rs2770102	−0.18	0.014	LINC00340
EE	rs11583200	0.19	0.015	ELAVL4
EE	rs2228213	−0.13	0.021	HIVEP1
EE	rs1205	0.12	0.021	CRP
EE	rs2845885	0.12	0.028	MACROD1
EE	rs3093059	0.16	0.03	CRP

**Table 4:** Descriptive statistics for GRS distribution and *p*-values for normality test. The mean, median, minimum, and maximum values of the GRS distribution for each category (CE, effectiveness of carbohydrate intake changes; FE, effectiveness of fat intake changes; TE, effectiveness of total calories intake changes; EE, effectiveness of exercise) are shown. All four GRS distributions followed a normal distribution based on Shapiro-Wilk test.

	CE	FE	TC	EE
Mean (SD)	0.43 (3.8)	1.4 (2.5)	5.4 (4.2)	1.5 (3.1)
Median	0	1	5	2
Minimum	−13	−9	−10	−9
Maximum	15	11	30	11
*p*-value for normality test	<2.2 × 10^−^^16^	<2.2 × 10^−^^16^	6.874 × 10^−^^15^	7.203 × 10^−^^14^

## Data Availability

Data are owned by the Korea Center for Diseases Control and Prevention (KCDC) and are available after approval from the committee of KCDC (http://cdc.go.kr/; admin@koreabiobank.re.kr).

## Supplementary Materials

The following are available online at http://www.mdpi.com/2072-6643/10/3/266/s1, Figure S1: Venn diagram of the number of SNPs identified by the GLM and numbers of overlapping among the four categories; Figure S2: Minor allele frequencies of SNPs that influence the effectiveness of changes in carbohydrate intake (CE), effectiveness of changes in fat intake (FE), effectiveness of changes in total calorie intake, and effectiveness of exercise status (EE); Table S1: Gene names of the corresponding SNPs for each category. File: Summary of the selected SNPs for GRS calculation.
