# Supplementary material for: Impact of Genetic Variants on the Individual Potential for Body Fat Loss
Source: Nutrients. 2018 Feb 26;10(3):266. doi: 10.3390/nu10030266 (PMC5872684; doi:10.3390/nu10030266)
Supplement: Supplementary file 1 [file nutrients-10-00266-s001.zip › 1_Nutrients_Supplementary_Figure-Table_Cha_et_al_20171025.pdf]

SUPPLEMENTARY FIGURES AND TABLES

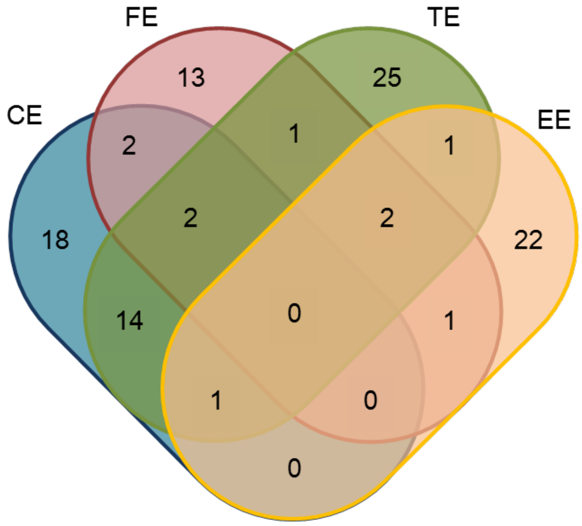

Supplementary Figure 1. Venn diagram of the number of SNPs identified by the GLM and numbers of overlapping among the four categories. After filtering the SNPs in the LD group, the final numbers of SNPs for each category are presented in the Venn diagram. CE and TE showed the most number of overlapping SNPs.

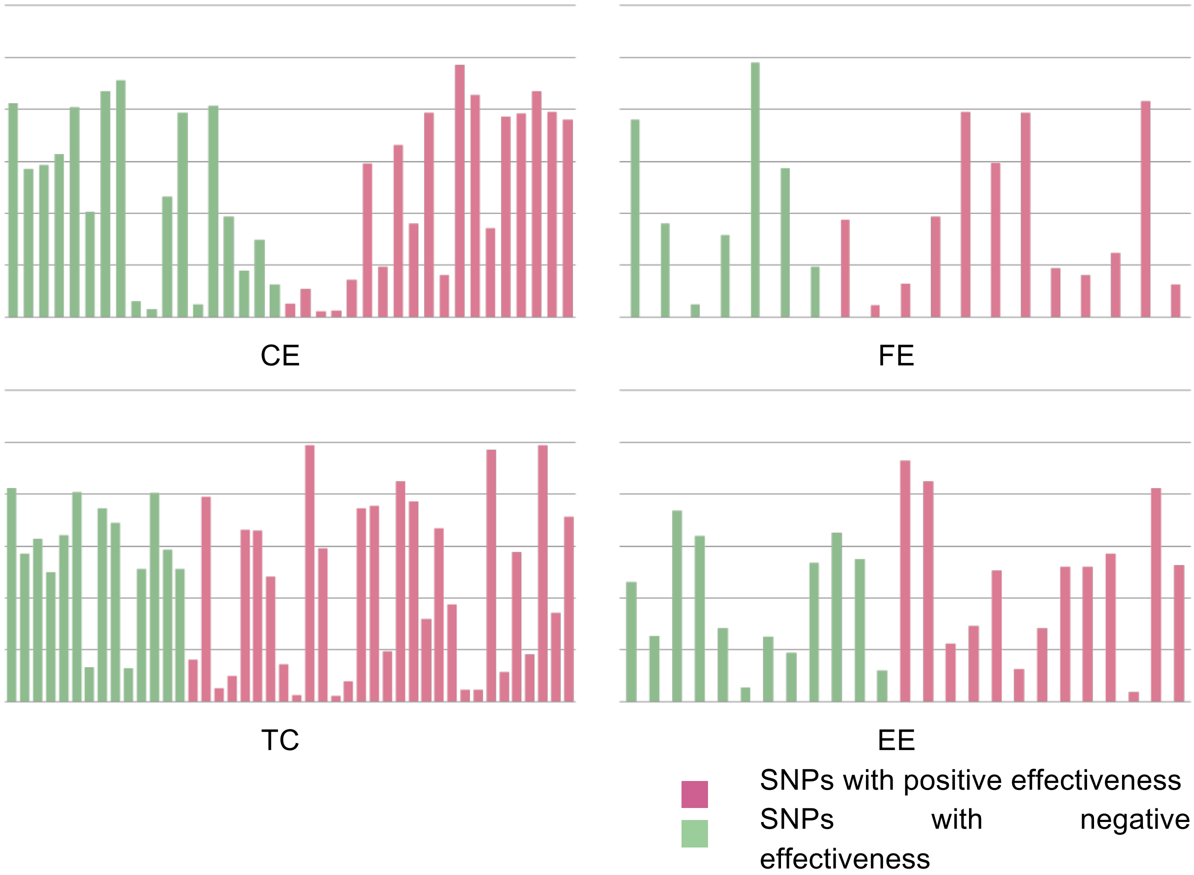

Supplementary Figure 2. Minor allele frequencies of SNPs that influence the effectiveness of changes in carbohydrate intake (CE), effectiveness of changes in fat intake (FE), effectiveness of changes in total calorie intake, and effectiveness of exercise status (EE). X-axis indicates the set of SNPs included in the final set of SNPs for scoring genetic risks. Y-axis indicates minor allele frequencies. SNPs with negative effects were colored dark blue, whereas those with positive effects were colored light blue.

CE	FE	TE	EE
AGT (2)	AGT (2)	AGT	ACAP2
ALK	ARAP1	ALK	ADCY5
APOA2	FAM212B	APOA2	AGBL4
ARAP1	FHIT	COL16A1	AGT (2)
CPNE4	intergenic (7)	COL25A1	APOA2 (2)
DKFZp686K1684	KCNH5	FAM19A2	ASB4
FAM19A2	KCNQ1	FLJ30838	BDNF-AS1
GLRA3	PLEKHG5	GGNBP2	CRP
intergenic (12)	PPARG (3)	HDAC9	CRP
KCNQ1	STK33	intergenic (18)	EIF2AK4
MRAS		LOC63930	ELAVL4
OSBPL10		OSBPL10	HIVEP1
PPARG (9)		PAX7	intergenic (4)
RPS10-NUDT3		PPARG (10)	KCNH5
SSBP3		RBFOX1	LINC00340
TRAF3		RPS10-NUDT3	MACROD1
USP37		tAKR	PPARG (2)
		ZNF131	RPS10-NUDT3
			SEC16B

Supplementary Table 1. Gene names of the corresponding SNPs for each category. Out of 46 SNPs in the CE group, 35 SNPs had corresponding matches to gene names, while 11 SNPs were intergenic. Of 16 SNPs for FE, 13 SNPs matched to a particular gene name, while three SNPs were mapped to intergenic regions. Out of 44 SNPs in the TE category, 26 SNPs were matched to 21 genes and 18 intergenic SNPs. Out of 25 SNPs in the EE category, 21 SNPs had corresponding gene names and four SNPs were intergenic.
